# The C8 Health Project: Design, Methods, and Participants

**DOI:** 10.1289/ehp.0800379

**Published:** 2009-07-13

**Authors:** Stephanie J. Frisbee, A. Paul Brooks, Arthur Maher, Patsy Flensborg, Susan Arnold, Tony Fletcher, Kyle Steenland, Anoop Shankar, Sarah S. Knox, Cecil Pollard, Joel A. Halverson, Verónica M. Vieira, Chuanfang Jin, Kevin M. Leyden, Alan M. Ducatman

**Affiliations:** 1 Department of Community Medicine and; 2 Center for Cardiovascular and Respiratory Sciences, West Virginia University School of Medicine, Morgantown, West Virginia, USA; 3 Brookmar, Inc., Parkersburg, West Virginia, USA; 4 Public Health and Environmental Research Unit, London School of Hygiene and Tropical Medicine, London, United Kingdom; 5 Department of Environmental and Occupational Health, Rollins School of Public Health, Emory University, Atlanta, Georgia, USA; 6 Department of Environmental Health, School of Public Health, Boston University, Boston, Massachusetts, USA; 7 Department of Political Science, Eberly College of Arts and Sciences, West Virginia University, Morgantown, West Virginia, USA

**Keywords:** C8, environmental contamination, perfluorocarbons, PFOA, toxic tort settlement

## Abstract

**Background:**

The C8 Health Project was created, authorized, and funded as part of the settlement agreement reached in the case of *Jack W. Leach, et al. v. E.I. du Pont de Nemours & Company* (no. 01-C-608 W.Va., Wood County Circuit Court, filed 10 April 2002). The settlement stemmed from the perfluorooctanoic acid (PFOA, or C8) contamination of drinking water in six water districts in two states near the DuPont Washington Works facility near Parkersburg, West Virginia.

**Objectives:**

This study reports on the methods and results from the C8 Health Project, a population study created to gather data that would allow class members to know their own PFOA levels and permit subsequent epidemiologic investigations.

**Methods:**

Final study participation was 69,030, enrolled over a 13-month period in 2005–2006. Extensive data were collected, including demographic data, medical diagnoses (both self-report and medical records review), clinical laboratory testing, and determination of serum concentrations of 10 perfluorocarbons (PFCs). Here we describe the processes used to collect, validate, and store these health data. We also describe survey participants and their serum PFC levels.

**Results:**

The population geometric mean for serum PFOA was 32.91 ng/mL, 500% higher than previously reported for a representative American population. Serum concentrations for perfluorohexane sulfonate and perfluorononanoic acid were elevated 39% and 73% respectively, whereas perfluorooctanesulfonate was present at levels similar to those in the U.S. population.

**Conclusions:**

This largest known population study of community PFC exposure permits new evaluations of associations between PFOA, in particular, and a range of health parameters. These will contribute to understanding of the biology of PFC exposure. The C8 Health Project also represents an unprecedented effort to gather basic data on an exposed population; its achievements and limitations can inform future legal settlements for populations exposed to environmental contaminants.

Perfluorooctanoatic acid (PFOA, or C8)is one member of the class of man-made perfluorocarbon (PFC) compounds. PFOA exists as an alkyl acid (PFOA), an ammonium salt [ammonium perfluorooctanoate (APFO)], or as a dissociated conjugate base [perfluorooctanoate (PFO)]. A closely related PFC is perfluorooctanesulfonate (PFOS; C8 sulfonate, or C8S). Additional, related PFCs include C5 [perfluoropentanoic acid (PFPeA)], C6 [perfluorohexanoic acid (PFHxA)], C6 sulfonate [perfluorohexane sulfonate (PFHS)], C7 [perfluoroheptanoic acid (PFHpA)], C9 [perfluorononanoic acid (PFNA)], C10 [perfluorodecanoic acid (PFDA)], C11 [perfluoroundecanoic acid (PFUnA)], and C12 [perfluorododecanoic acid (PFDoA)]. PFCs are used as plasticizers, wetting agents, and emulsifiers during the manufacture of fluoropolymers, including products that impart nonstick heat resistance to cookware or breathable yet waterproof properties to fabrics. PFCs may also result from the metabolism or environmental breakdown of fluorinated telomers, including chemicals used to coat commercial food packaging and for stain-resistant treatment for fabrics and clothing. PFOA may also be a residual impurity in personal care products.

## PFCs and health

PFOA and other PFCs persist in the environment and are found in groundwater and surface water worldwide (Yamashita et al. 2008). They are present in blood and other tissues of animal species throughout the world, including remote regions (Tao et al. 2006). Recent publications have extensively reviewed and summarized the known toxicologic properties, environmental distribution, and potential health concerns related to PFOA (Kennedy et al. 2004; Kudo and Kawashima 2003; Lau et al. 2007). Animal toxicology studies have suggested potential suppression of humoral immunity, neuroendocrine effects, and exposure-related gestational and developmental effects. Cumulative evidence from mammalian animal studies has suggested that the liver is an important target organ. Reported hepatotoxic effects include liver enlargement, hepatocellular adenomas, and peroxisome proliferation [specifically peroxisome proliferator-activated receptor (PPAR)-α], possibly suggesting a possible nongenotoxic carcinogenic mechanism for PFCs.

Additionally, combined evidence supports that PFCs generally, and PFOA and PFOS specifically, are present in the sera of diverse human populations (Lau et al. 2007). In the United States, almost all National Health and Nutrition Examination Survey (NHANES) samples contained these chemicals, with a U.S. population median of 5 ppb PFOA. Both PFOA and PFOS concentrations were higher in the serum of men and those with higher education (Calafat et al. 2007a, 2007b). Although potential sources of human exposure continue to be investigated, current, known sources of PFOA exposure generally include drinking water, household dust, and food or migration from food packaging (in particular, commercial and fast-food/take-out packages) and cookware (Lau et al. 2007). Occupational studies have shown elevated worker exposures in manufacturing processes that use PFOA or PFOS (Emmett et al. 2006; Olsen et al. 2003).

Human population studies, predominantly medical surveillance studies of male American workers exposed occupationally to PFOA or PFOS, have reported inconsistent findings (Lau et al. 2007). Although some studies have reported associations between exposure and cancer (bladder and prostate in particular), lipids, liver enzymes, and some thyroid hormones, follow-up studies and others have either contradicted earlier findings or found evidence suggesting explanation through confounding parameters (Lau et al. 2007). Maternal serum and neonatal cord blood studies have implicated an association of PFOA or PFOS with birth weight (Apelberg et al. 2007; Washino et al. 2009), but there are also contradictory findings in high-exposure populations (Nolan et al. 2008). The half-life of PFOS and PFOA in human sera has been reported as approximately 5 and 3.5 years, respectively (Olsen et al. 2007).

In a review process that remains ongoing, the U.S. Environmental Protection Agency (EPA) is considering evidence and classification of PFOA as a likely human carcinogen (U.S. EPA 2006). The U.S. EPA’s PFOA Stewardship Program proscribes PFOA, PFOA precursors, and related, higher homologue chemicals from emissions and products by 2015 (U.S. EPA 2009). In the European Union, the use of PFOS and derivatives was stopped in 2000 and banned in 2008, although PFOA use remains largely unregulated (Jensen and Leffers 2008).

## Origin of the C8 Health Project

The C8 Health Project can be traced to legal actions taken by a local family. A portion of this family’s farmland was sold to DuPont (E.I. du Pont de Nemours & Co.) in 1984, which subsequently converted the land parcel into a site to dispose of waste products from PFC manufacturing from their Washington Works plant. The family alleged that the (then unknown) chemicals from the landfill were responsible for family illness, wildlife death, and the death of almost 300 head of their cattle [see Supplemental Material, Note 1 (doi:10.1289/ehp.0800379.S1 via http://dx.doi.org)]. The family agreed to a confidential settlement with DuPont in 2001; associated legal activities, including independently commissioned studies and reports filed by government agencies, served to provide environmental data and to heighten local awareness of the exposure, coincident with an emerging scientific literature.

The following points summarize key events in the almost two-decade time line leading to the C8 Health Project:

- Cumulative evidence detected PFOA contamination of water supplies along the mid-Ohio River Valley (approximately 1984–2004). Water pollution was attributed to direct industrial releases from DuPont’s Washington Works plant into the Ohio River, a principal source of public drinking water, and airborne pollution more broadly contaminated water tables and aquifer systems, with subsequent contamination of well water, an important drinking water source in a rural community [see Supplemental Material, Note 2 (doi:10.1289/ehp.0800379.S1)].
- August 2001 through April 2002: Thirteen plaintiffs filed a lawsuit against DuPont, which was subsequently certified as a class action, *Jack W. Leach v. E.I. du Pont de Nemours & Co.* (Civil Action No. 01-C-608), filed in the Wood County, West Virginia, Circuit Court. The “Class” was defined as individuals, in West Virginia or Ohio, whose drinking water had been contaminated by quantifiable levels of PFOA.
- November 2004: A multicomponent $107 million pretrial settlement between the Class and DuPont was reached. Complete settlement terms are part of the public record. Key provisions included the following: a $70 million award for Class members, of which $20 million was required to be used for health and education projects; provision of water treatment technologies to remove PFOA from the water supply of the six affected water districts; and formation of an independent panel of three scientific experts to carry out a community study and determine if there is a “probable link” [see Supplemental Material, Note 3 (doi:10.1289/ehp.0800379.S1)] between PFOA exposure and human disease.

The settlement broadly outlined terms of agreement but did not detail how they were to be satisfied. Post hoc negotiations between settling parties resolved that the health and education projects and Class payments would be achieved through a population-wide health study of the Class, initially known as the “Settlement Class Health & Education Project” and later the “C8 Health Project” (the Project). An independent company, Brookmar, Inc., was created to design, publicize, and implement the Project under court supervision. Three epidemiologists (the C8 Science Panel) were appointed to determine the presence or absence of what the court termed a “probable link” between PFOA exposure and human disease [see Supplemental Material, Note 3 (doi:10.1289/ehp.0800379.S1)]. In addition to Project data, the C8 Science Panel is to include data de novo, prospective community studies that they proposed and are conducting.

The Project faced significant implementation challenges, including Court and population expectations for rapid time lines as well as absence of precedent for the likely scale of the community project. Project participation was the established route for Class members to benefit from the settlement, but neither Class size nor participation was known *a priori*. Accordingly, Brookmar, Inc. developed procedures to accommodate rurality, shift workers, eligible Class members no longer living in the area, a wide range in participant age and mobility, a deliberately short survey period, community apprehension expressed regarding data privacy and concern about adverse effects on insurability and even employability, and the desire for participants to receive personalized information about laboratory results and general information about Project findings. The data collection methodology implemented by Brookmar, Inc., agreed to implicitly or explicitly by counsel for the settling parties, is described below.

## C8 Health Project Methods

### Eligibility

Class eligibility was defined by exposure to contaminated water, a combination of geographic and concentration criteria, and exposure duration. Key criteria included *a*) exposure to contaminated water from any of six public water districts [two in West Virginia, four in Ohio; see Supplemental Material, Figure 1 (doi:10.1289/ehp.0800379.S1)] or from private water sources within the geographical boundaries of the public water sources which contained ≥ 0.05 ppb PFOA, and *b*) the ability to document a minimum 12 months of exposure to contaminated water between 1950 and 3 December 2004, at primary residence, place of employment, or school.

Participants supplied documentation demonstrating both their identity and exposure using a combination of Court-defined acceptable documents. Brookmar, Inc. independently verified the authenticity of documents with the issuing agency, and identity documentation was examined to ensure that participants were enrolled only once. Scanned document copies became part of the participant’s Project electronic data record.

### Data components

Four types of data were collected: a health survey, self-reported anthropometric measurements, a blood sample, and a medical chart review to validate selected self-reported diagnoses. In identifying clinical laboratory tests and selecting diagnoses for validation, priority was given to those with potential associations with PFC exposure as reported in the scientific literature. Clinical laboratory tests included serum lipid, immune, and inflammatory markers; liver, kidney, and thyroid function; complete blood count; serum electrolytes and protein; and endocrine function, including insulin and glucose [see Supplemental Material, Note 4 (doi:10.1289/ehp.0800379.S1)]. Validated medical diagnoses included heart disease, cancers, thyroid disease, neurologic disorders, inflammatory and autoimmune disorders, and pregnancy complications [see Supplemental Material, Note 5 (doi:10.1289/ehp.0800379.S1)].

The health survey gathered demographic data; current and historic residential and employment information, including water source and use; personal medical diagnoses, treatments including medications, and physical symptoms; family medical history; pregnancy history and pregnancy-related outcomes for women; and information about lifestyle and health behaviors. Participants also self-reported their own height, weight, and blood pressure. Brookmar, Inc. contracted with a separate company to independently pilot test the survey, and revisions were made based on pilot-test findings. The final version of the survey was accepted by the settling parties. The survey, a list of the clinical laboratory tests, and the 18 medical diagnoses verified by medical record review are publically available on The C8 Health Project WVU Data Hosting Website (C8 Health Project 2009).

### Enrollment

An independent information technology (IT) company was contracted to build and manage informatics solutions that addressed Class security concerns and created a web-based mechanism for Project registration and health survey completion. Participants could alternatively register in person and use paper-based surveys. After registration and completion of the health survey, participants received instructions regarding requirements for demonstrating eligibility and making an appointment at a Project data-collection site. Standard data quality-assurance techniques for survey data, including a quota for data duplicate entry for paper-based surveys and electronic-based logic rules (e.g., limited-answer menus) for Web-based surveys, were in place for the health survey.

### Data collection procedures

Temporary modular office units were established in each water district, staffed with nurses, phlebotomists, and intake personnel, and equipped for venipuncture, blood processing, and short-term record and blood sample storage. Participants could schedule appointments between 0730 and 1930 hours at the location of their convenience. Because of both feasibility and participant considerations, fasting was not required for phlebotomy, although self-reported fast duration was collected to facilitate interpretation of laboratory results.

At in-person appointments, participants submitted eligibility documentation and the water district indicated by the exposure documentation provided was recorded; this was usually but not always the source of greatest exposure. Project staff verified demographic data, current residential information, and completion of the health survey and asked participants to report their current height, weight, and blood pressure. Participants voluntarily submitted a blood sample.

Each verified participant received $150 for completing the health survey and an additional $250 for providing a blood sample (regardless of sample quantity or quality). The payment amount reflected the compensation intentions of the settlement and remuneration for Project participation expenses.

### Blood sample processing and laboratory methods

Blood samples were obtained and processed at individual data collection sites. Samples were drawn into four tubes per participant, with a maximum 35 mL for adults and 26 mL for children. Tubes were spun, aliquotted, and refrigerated until shipping. For limited-volume samples, serum was aliquotted with priority for PFC analysis. Samples were shipped on dry ice daily from each data collection site to the laboratory retained to measure serum PFCs. The clinical laboratory contracted to perform the clinical chemistry analysis picked up samples daily from each data-collection site. Additionally, an aliquot of serum from each participant was frozen and subsequently stored in a Project tissue bank.

Clinical laboratory tests were performed at a large, independent, accredited clinical diagnostic laboratory (LabCorp, Inc., Burlington, NC, USA). A customized health level 7 interface generated immediate, on-site laboratory-specific identification numbers and tube labels and permitted subsequent electronic transfer of clinical laboratory results directly into the Project data system. Clinical laboratory tests and quality assurance were performed in accordance with the accreditation standards required of this laboratory.

The primary laboratory performing PFC analysis (Exygen Research Inc., State College, PA, USA) was selected based on its ability to meet U.S. Food and Drug Administration guidelines for bioanalytical method validation, a lower limit of quantification of 0.5 ng/mL, and 96-well-plate–based technology allowing for high-throughput capability. This was also the laboratory of record for a previously reported, independently performed study of residents in one water district included in the Project (Emmett et al. 2006). The PFOA quantification and validation methodology used by this laboratory has been previously detailed (Flaherty et al. 2005). The analytic protocol used for the Project was a modification of this methodology. Briefly, the technique used a protein precipitation extraction together with reverse-phase high-performance liquid chromatography/tandem mass spectrometry. Spectrometric detection was performed using a triple quadrupole mass spectrometer in selected reaction monitoring mode, monitoring for the individual *m*/*z* transitions for each of the 10 PFCs and the ^13^C-PFOA surrogate. Results for the 10 PFCs measured were incorporated into the Project information system through a Windows-based program [see Supplemental Material, Note 6 (doi:10.1289/ehp.0800379.S1)].

### PFC quality assurance

A two-tiered quality assurance program was implemented consisting of *a*) evaluation of test reliability in the primary lab (intralab reliability) with the use of blank samples, samples spiked with a known PFC concentration, and participant duplicate samples, and *b*) use of a second, external laboratory (AXYS Analytical Services Ltd., Sidney, BC, Canada) to determine PFC concentrations for participant duplicate samples (interlab reliability). This laboratory, with the ability to monitor 10 individual PFCs and a lower limit of quantification of 0.2 ng/mL, employed analytic methods previously described (Kuklenyik et al. 2004; Taniyasu et al. 2005). Briefly, the technique used solid-phase extraction on a weak anion-exchange column followed by reverse-phase high-performance liquid chromatography/mass spectrometry. Spectrometric detection was performed using a triple quadrupole mass spectrometer in selected reaction monitoring mode, monitoring individual *m*/*z* transitions for each of the target PFCs, the ^13^C-PFOA, ^13^C-PFOS, and ^13^C-PFDA surrogates and the ^13^C-PFOA and FOUEA (^13^C-2H-perfluoro-2-decenoic acid) instrument internal standards.

To assess method performance at the primary laboratory, quality control samples in the form of two control serum blanks, two lab control spikes in control serum, and two sample duplicates were included with each batch of 90 samples analyzed. ^13^C-PFOA (surrogate) was also added to every sample before extraction to assess lab preparation. Bulk control blanks and spikes were prepared at the primary lab and sent to the sampling sites. They were then blindly returned with every shipment of samples for analysis to assess storage, transport, and laboratory preparation effects. For these quality control samples, the Project IT system generated in-line dummy identification numbers and two sets of lab-ready, bar-coded phlebotomy tube labels. Site nurses aliquotted two sets of sample tubes, and both were included as part of the standard shipment to the primary laboratory. Based on a data collection site-specific sampling plan, samples were also automatically identified by the Project IT system for the secondary lab. Labels and tubes were generated similarly, as were sample aliquots. Results from quality assurance samples were segregated from the main, participant database post hoc by the IT company, the only group unblinded to identification numbering.

During analysis of quality assurance results, a consistent difference between the primary and secondary laboratory was detected (~ 30%) for samples obtained during the first 4 months of the Project. Investigation and additional, targeted intra- and interlab retesting confirmed these directional (higher) differences. Per a court filing, Exygen discussed the cause as a problem of initially prepared samples used for internal calibration [see Supplemental Material, Note 7 (doi:10.1289/ehp.0800379.S1)]. Affected samples (~ 25,000) were retested using serum stored in the Project tissue bank; quality assurance testing, including sample duplicates and replaced spiked and calibration samples, was also repeated. Retested results demonstrated a consistent decrease from initial results and increased consistency with the secondary lab. All analytic results presented here include only retested values for those affected serum samples.

For quantitative assessment of quality assurance test results, final test values were matched to participant quality assurance values for the primary or secondary lab. Results reported as less than the limit of detection (LOD) were treated conservatively and excluded from quality assurance analyses. Agreement between two measures was assessed with the absolute difference, percent difference (absolute difference between values divided by value means), and coefficient of variation (SD divided by mean), which were then summarized (mean and median) across the matched-samples results.

### Validation of select medical diagnoses

Participants self-reporting one of the 18 targeted diagnoses were asked to provide the time and location of diagnosis. After obtaining appropriate record release consent, Brookmar, Inc., requested a copy of supporting documentation from a medical record or pathology report from health care providers. Cooperating providers were compensated $10 per necessary page. For approximately 36,000 validated diagnoses, the following was recorded: self-reported diagnosis; support (confirmation), nonsupport (negation), missing (records not obtained), or substitution (i.e., documentation supported a different diagnosis); the alternate diagnosis where appropriate; and type of documentation used for verification. Review of medical records and determination of diagnostic verification were performed by nurses employed by Brookmar, Inc.

### Consenting procedures

Brookmar, Inc., required that participants read (and “check” affirmatively) an introductory section of the health survey that explained the purpose and procedures of the Project, and risks and benefits of participation. This language is publically available as part of the survey tool. All participants submitting a voluntary blood sample completed the standard consent and release forms of the clinical laboratory contracted for phlebotomy. Brookmar, Inc. obtained a separate consent form for the release of medical records necessary for diagnosis validation, which was subsequently mailed (along with a cover letter and specific documentation request) to the health care provider identified by the participant.

The Project group at West Virginia University and the C8 Science Panel obtained institutional review board (IRB) approval from their own institutions permitting access to deidentified Project data. With assistance from Brookmar, Inc., the C8 Science Panel obtained additional IRB approval allowing access to identified data, which facilitates contacting participants for enrollment in follow-up studies.

### Implementation

After input by the settling parties, Brookmar, Inc. used multiple avenues to publicize the Project. Communications about Project eligibility requirements, enrollment, data collection procedures, time line, and remuneration included a series of open meetings conducted in five of the six water districts, Project Web site messages, a phone bank, and press conferences with local media, an important source of information throughout the duration of the Project.

Brookmar, Inc. also maintained communication with local health care providers. Meetings were conducted to inform the medical community about Project procedures, including medical record requests, as well as the information that participants would be provided pursuant to their Project participation.

Efforts to ensure full access for interested participants included multiple sites, 12-hr daily appointment availability, and disability accommodation including handicap access and assistance completing the health survey. Brookmar, Inc. also coordinated remote data collection for those otherwise eligible Class members unable to travel to a data collection site (e.g., those no longer living in the vicinity). For these participants, eligibility documentation was submitted via mail, followed by remote completion of the health survey and a personal telephone interview. These remote participants then completed phlebotomy at an identified, local, accredited laboratory. Of the total participants, approximately 600 participated via these procedures (Flensborg P, personal communication).

Brookmar, Inc. was unable to accommodate participants cognitively unable to complete the survey (and without a representative to accurately complete it on their behalf) or those physically unable to travel to a phlebotomy site. Thus, these groups are likely underrepresented among Project participants.

### Data analysis and reporting

Participants were mailed individualized results for clinical lab tests, including laboratory normal ranges and flags for abnormal findings. For severely abnormal values, emergency flags triggered a personal communication from Brookmar, Inc. personnel with advice to seek prompt medical attention. Participants also received a report of their PFC values.

Upon completion of the Project, Brookmar, Inc. filed an electronic data set with the Wood County Court in May 2008. The data set included the health survey, clinical laboratory and PFC values, an image of eligibility documents, and record of payment. To protect participant privacy, the presiding judge subsequently sealed the data set. A mechanism is currently being sought wherein an agency, likely of the federal government, would maintain and make accessible a deidentified data set for public research use.

The C8 Science Panel is conducting analyses using the Project cross-sectional data collected and is also conducting its own independent, environmental and population-based studies, also financed by the settlement. For consenting participants, the C8 Science Panel is able to link Project-collected data with follow-up and longitudinal studies. A description of ongoing studies is available on the C8 Science Panel’s web site (C8 Science Panel 2009).

A tissue bank of participant frozen serum was established at West Virginia University in which samples are stored, handled, and accessed in a manner consistent with the IRB protocol governing the West Virginia University Tissue Bank. The sera can be linked to deidentified Project data and may be used for further studies related to human PFC physiology.

Brookmar, Inc. contracted with the West Virginia University School of Medicine to report to the general public descriptive summaries of results from the Project. Summary data are reviewable at a web site established for that purpose (C8 Health Project 2009).

The C8 Science Panel and the West Virginia University Project group are preparing and submitting analyses of associations between PFCs and health outcomes, intended for peer-reviewed journals. These will form part of the evidence that will assist the C8 Science Panel in meeting their Court-appointed obligation to determine “probable links” [see Supplemental Material, Note 3 (doi:10.1289/ehp.0800379.S1)] between PFOA exposure and health outcomes.

### Data cleaning

In the first phase of data cleaning, the IT company ensured that question responses were consistent with question “skip patterns” and menu options, as well as ensuring consistent coding and formatting for question responses. For the deidentified data set, text fields were scrubbed to eliminate potentially identifying information. In the second phase of data cleaning, completed collaboratively by the C8 Science Panel and West Virginia University Project team, continuous variables were examined and decision rules were created for outliers and missing values. Lab-generated error messages for samples that could not be analyzed were deleted and results set to “null.” For clinical lab results either lower or higher than the LOD, values were replaced with 50% below or above the lower or upper LOD, respectively.

Though serum samples were analyzed for 10 PFCs, not all PFCs were detectable in all samples tested. Four PFCs (PFHS, PFOA, PFOS, PFNA) were detectable in almost all (> 97%) samples; for these PFCs, test results reported as less than the LOD were substituted with 0.25 ng/mL (50% of the lower LOD of 0.5 ng/mL). Three PFCs (PFHxA, PFHpA, PFDA) were detectable in approximately 50% of the samples; results for these PFCs are reported with and/or without substitution for values reported as less than the LOD. Three PFCs (PFPeA, PFUnA, PFDoA) were detectable in only a negligible portion of the tested samples and are not reported here or included in further analyses. Thus, results reported here include 7 of the 10 tested PFCs (PFHS, PFOA, PFOS, PFNA, PFHxA, PFHpA, PFDA).

### Estimation of participation rates

Total Project participation was estimated by water district as the number of participating residents divided by the total contemporaneous population in that water district. Water district population was estimated using 2005–2006 Census population estimates for block groups, the smallest Census geographic unit that could be accurately identified. Block groups intersecting with water districts were determined, and the population of each block group was apportioned to the water district based on the ratio of water district area to block group area within each block group. The number of participants resident in each water district was based on the assigned water district, that for which the participant presented qualifying eligibility documents, and self-reported residence at the time of enrollment. Participation estimates for each water district was estimated for age and gender strata.

## Results

Project enrollment totaled 69,030. Approximately 80% completed registration and the health survey online (Flensborg P, personal communication). PFC and clinical laboratory analyses were available for > 65,000 participants. Although children < 10 years of age had highest proportion without available blood analyses (almost one-third), laboratory data were nevertheless available for > 3,400 of these children. Consistent with regional demography, > 97% of participants identified themselves as white, and educational attainment and income levels were lower than national averages. Participants ranged in age from 1.5 years to > 100 years, with a mean ± SD age of 39.1 ± 19.9 years [see Supplemental Material, Table 1 (doi:10.1289/ehp.0800379.S1)].

Twenty-six percent of adults (≥ 18 years of age at enrollment) reported current smoking, and an additional 26% reported former smoking. A substantial proportion of children (63.3%) and adults (69.1%) lacked a regular exercise program. Further, 39% of children were classified as being at-risk or already overweight based on body mass index (BMI) percentile, and 69% of adults were classified as overweight or obese based on BMI. However, because BMI calculations were completed using self-reported height and weight, they may underestimate actual population proportions. These demographic and health risk data are generally consistent with estimates for Appalachia from nationally representative data sources (Halverson et al. 2004) [see Supplemental Material, Table 2 (doi:10.1289/ehp.0800379.S1)].

At the time of enrollment, most participants reported current residence in Ohio (52%) or West Virginia (45%); 63% of participants were resident in a qualifying water district at the time of their participation. Average monthly enrollment in the Project was 5,310, with enrollment peaking in January 2006 (8,003 participants). The largest proportion of participants qualified through Lubeck Public Service District (24.6%), followed by Tuppers Plains (20.4%) [see Supplemental Material, Table 3 (doi:10.1289/ehp.0800379.S1)].

As shown in Table 1, an estimated 80.3% of the population resident in the water districts during the enrollment period participated in the Project, with a slightly higher proportion of women compared with men participating. Participation by water district ranged from 70.6% of the resident population in Tuppers Plains to almost 92% in the Village of Pomeroy. The age groups with the lowest estimated participation were the elderly (> 80 years of age) and young children (< 4 years of age).

Serum analysis results for 10 PFCs (PFPeA, PFHxA, PFHS, PFHpA, PFOA, PFOS, PFNA, PFDA, PFUnA, PFDoA) were available for 66,899 participants. Table 2 summarizes the proportion of tested samples with a detectable PFC concentration and the number of samples for which 50% of the lower LOD was substituted.

**Figure 1:**
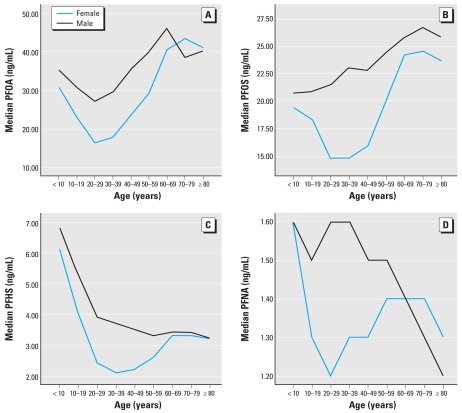
PFC concentrations stratified by age and sex: (*A*) PFOA, (*B*) PFOS, (*C*) PFHS, and (*D*) PFNA.

Table 3 reports population summary statistics for the seven PFCs, stratified by sex and age groups. Because of population homogeneity, stratification by ethnicity was not performed. Figure 1 highlights patterns by age and sex in the four widely detectable PFCs (PFHS, PFOA, PFOS, PFNA). For each, median concentrations were higher in males in most age groups. For PFOA and PFOS (Figure 1A,B), population median concentrations demonstrated a J-shaped pattern, with higher values in younger age groups, lowest values in young- to middle-adult age groups, and highest population concentrations in older adult age groups. For PFHS and PFNA (Figure 1C,D), the highest population median concentrations were observed in children.

**Figure 2:**
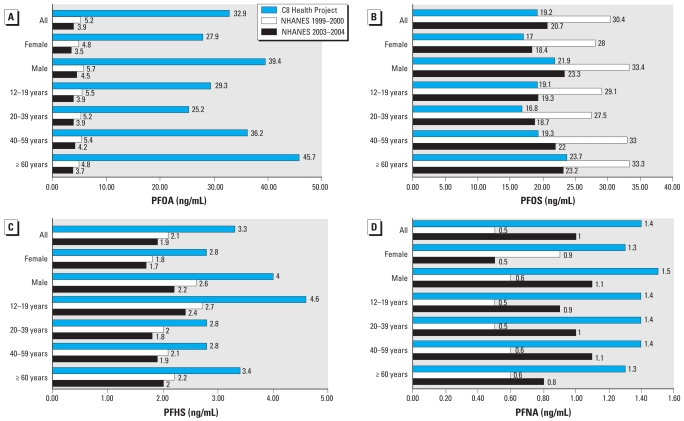
Geometric means (ng/mL) for PFC serum concentrations for C8 Health Project results [versus two NHANES samples (2003–2004–1999–2000)]: PFOA (*A*), PFOS (*B*), PFHS (*C*), and PFNA (*D*).

Figure 2 compares Project results for these same four PFCs with results from two nationally representative NHANES samples. An important difference between NHANES and Project results is the inclusion of children < 12 years of age in the Project, although this Project age group is likely to have a minimal impact on overall population results in a sample size > 65,000. For all PFCs except PFOS, serum concentrations reported for the Project exceeded NHANES results. The largest differences were observed for PFOA, where the Project population had more than a 500% (1999–2000) or 700% (2003–2004) larger geometric mean. Smaller but still substantial differences were also observed for PFHS [57.1% (1999–2000) and 73.6% (2003–2004) larger] and PFNA [178% (1999–2000) and 39% (2003–2004) larger]. In contrast, the Project population had a 36.8% (1999–2000) and 7.1% (2003–2004) lower geometric mean for PFOS.

Unadjusted, nonparametric Spearman’s rho rank-order correlation analysis was performed for 21 pairings (seven PFCs) for the overall population. In general, correlations between the PFCs were low. Modest correlations (~ 30%) were observed between the pairings of PFHS–PFOA, PFHS–PFNA, PFOA–PFOS, and PFOS–PFDA. Larger correlations were observed between PFHpA and PFOA (~ 40%), PFHS and PFOS (~ 50%), PFOS and PFNA (~ 50%), and PFNA and PFDA (~ 60%). No discernable differences in correlations patterns were observed between adults and children or between sexes [data not shown; see Supplemental Material, Figure 2 (doi:10.1289/ehp.0800379.S1)].

Table 4 reports serum PFC concentrations, weighted by age and sex, stratified by qualifying water district. Participants in Little Hocking Water Association had the highest levels of PFOA, > 70% higher than the next group, participants with private wells. For all PFCs other than PFOA, the 157 participants qualifying through contaminated private wells had the highest serum concentrations. Participants in Lubeck Public Service District had the highest reported values for PFHxA, PFOS, PFNA, and PFDA. The highest values of PFHS were found in participants in the City of Belpre, Ohio.

Table 5 summarizes results for the quality assurance analysis. The highest agreements, both intralab (within the primary lab) and interlab (between the primary and secondary lab), were observed for PFHS, PFOA, PFOS, and PFNA. The median intralab difference for PFOA and PFOS was 5.5% and 4.06%, respectively, whereas the median interlab difference for PFOA and PFOS was 16.7% and 14.01%, respectively. The least agreement, either intra- or interlab agreement was observed for PFHxA. For PFHpA and PFDA, intralab but not interlab agreement was observed.

## Discussion

This unique and large survey resulted from the pretrial settlement agreement of a class action lawsuit and a court-supervised health study of a population (*n* = 69,030) exposed to sustained environmental contamination with PFOA. The demographic characteristics and health behaviors of the participants reflect that of the affected, mostly rural, Appalachian communities: predominately white, with levels of education and income lower than the national average, and higher rates of obesity and other health risk behaviors such as smoking and inactivity. Challenges posed by the definition of the Class, specifically the prolonged (50-year) exposure period and inclusion of residential, employment, and school-based exposure, preclude an exact determination of the total eligible population. However, a reasonable approximation suggests that 80% of the current population in affected water districts participated in the Project, although 37% of Project participants resided outside an affected water district at the time of their Project enrollment. It is probable that a combination of public concern about chemical contamination and monetary compensation contributed to participation. The high rates of population participation and the high rate (estimated at 80%) of online (vs. paper survey) enrollment and completion of the health survey in a rural, poor area may provide useful lessons for future population surveys.

As anticipated, study serum concentrations of PFOA, the identified environmental contaminant, deviated markedly from those of a representative, national sample in all affected water districts. The unadjusted population geometric mean of 28.2 (median, 32.91) ng/mL was 6- to 8-fold higher than nationally representative values from the NHANES study. However, observations of serum concentrations for PFHS and PFNA that were higher than national samples but concentrations for PFOS lower than national samples were not anticipated. The distribution and pattern of PFHS and PFNA concentrations is not similar to that of PFOA, suggesting a possible exposure source other than the facility identified as the source of PFOA exposure. Comparisons between these two studies are interpreted within the context of possible differences in laboratory analytic techniques (although both studies used solid-phase extraction followed by high-performance liquid chromatography/tandem mass spectrometry) and measurement accuracy across a broad spectrum of concentrations.

Discernible sex differences for PFOA serum levels in most age groups are consistent with national findings in less exposed populations. The findings of higher serum concentrations of PFCs in children, particularly for PFHS and PFNA, warrant further study. Although these findings are unadjusted for potential confounders, higher concentrations in the youngest age groups is counter to traditional expectations of biologic burden paralleling cumulative environmental exposure.

The observation that the highest population burden of PFOA was found in the Little Hocking Water Association district is consistent with water quality measurement reports from the affected water districts [many of which are publically available; also see Supplemental Material, Note 2 (doi:10.1289/ehp.0800379.S1)]. However, the population PFOA results for Little Hocking are substantially lower than those previously reported in a smaller sample of Little Hocking residents (age- and sex-adjusted mean of 228 ng/mL reported here vs. 448 ng/mL; Emmett et al. 2006). Although the reasons for these differences are not clear, representativeness of the samples and study time periods are possible explanations. The same laboratory performed PFOA analysis in each study.

Although the highest serum concentrations of PFOA were observed in Little Hocking, it is interesting that higher burdens of other PFCs were found in other water districts. Investigating the association between water PFC concentrations and serum PFC concentrations and investigating the distributions of occupational contributions are beyond the scope of the present report. Further work is under way to model occupational, temporal, spatial, and temporal–spatial variation in water PFC levels, including differences between public water supplies and private wells and the association with serum PFC levels.

Correlations between the different PFCs were generally modest and without discernable age or sex patterns. Presently, the meaning of these correlations are unclear and will likely need to be interpreted later, within the context of a better understanding of patterns of cumulative exposure, environmental accumulation, and physiologic metabolism of these chemicals across the life span.

Previous studies have reported on the complexity of PFC determination (van Leeuwen et al. 2006). Although intralab measurements demonstrated reasonable stability, interlab measurements showed larger divergence. It is not surprising that measurements from the Project, which at times processed > 7,000 samples per month, would not achieve the same level of accuracy as federally funded projects with smaller sample sizes and smaller ranges of exposure. Spearman testing of quality-assurance results demonstrates that rank order is highly preserved for intra- and interlaboratory comparisons.

The large size of this cohort, together with the broad range of serum PFC values, provides unique opportunities for investigating associations between PFCs and human health. Although the prevalence study design inherently limits causal inference, the Project remains the largest and broadest study to date of associations between PFC exposure and human health. Subcohorts from this population, to be followed over time, have been identified and the C8 Science Panel has already begun enrollment in longitudinal studies. A multivariable analysis of factors determining PFOA levels in the population has been conducted and submitted for publication (Steenland et al. 2009). Additional targeted analyses investigating associations between PFCs and specific clinical chemistries and disease endpoints are already under way and will be reported elsewhere.

## Conclusion

The data from the 69,030 C8 Health Project participants provide valuable information on serum PFC concentration, demographic factors, clinical chemistry and self-reported disease in a population with a high participation rate. Ongoing work investigating the interrelationships between them will provide clues about possible etiologic relationships, within the limitations of the prevalence study design. In addition, they provide a valuable baseline characterization of this population for subsequent, prospective studies.

The results, therefore, have the potential to improve the current understanding of the biology of PFC exposure and are unprecedented among toxic tort settlements, most of which simply provide compensation for the Class without attempting to generate useful health and exposure data or assess health effects. As an innovative effort to gather data on an exposed population, the C8 Health Project can also serve as a model for future legal settlements for populations exposed involuntarily to environmental contaminants.

## Figures and Tables

**Table 1:** Estimated percent participation by water district.

Participants	City of Belpre (Ohio)	Tuppers Plains (Ohio)	Little Hocking Water Association (Ohio)	Lubeck Public Service District (West Virginia)	Mason County (West Virginia)	Village of Pomeroy (Ohio)	Total
Age group (years)							
0–4	39.6	54.2	54.8	38.5	39.5	36.2	45.5
5–10	79.8	71.7	81.8	72.3	79.8	87.7	77.0
11–14	85.6	73.7	90.7	92.2	95.8	88.6	87.2
15–19	101.9	82.0	90.7	107.5	96.9	110.2	94.6
20–24	99.6	72.5	77.6	81.0	90.0	98.9	82.8
25–29	88.2	74.6	84.5	73.2	90.5	86.7	81.9
30–34	87.6	76.1	92.9	81.6	93.7	92.7	86.1
35–39	87.3	76.7	90.8	94.5	94.6	103.2	88.7
40–44	94.8	75.7	86.2	100.1	91.4	94.9	88.4
45–49	94.6	71.3	85.0	89.6	90.8	109.7	85.4
50–54	85.8	79.9	84.8	79.1	83.3	102.5	82.8
55–59	89.3	69.6	84.1	89.5	78.1	98.8	81.2
60–64	98.3	71.3	82.2	96.8	82.8	101.6	85.1
65–69	89.4	67.7	88.8	91.3	78.8	88.6	82.0
70–74	90.5	57.7	78.6	84.4	74.0	94.3	75.6
75–79	79.8	54.2	55.2	65.1	58.3	82.6	62.1
≥ 80	51.8	35.4	41.9	39.8	31.0	63.2	40.1
Total	85.6	70.6	82.3	83.9	82.3	91.6	80.3
Total ≥ 20	87.8	70.2	82.6	85.2	83.1	94.8	81.0
Sex							
Male	85.0	69.6	80.9	82.8	79.9	89.2	78.8
Female	86.1	71.6	83.8	85.0	84.8	93.6	81.8

**Table 2:** Availability of detectable serum concentrations for PFCs shown as the number (%) of samples.

PFC	No. (%)with detectable concentration	No. (%)with concentration < LOD	No. (%)with substitution of 50% of LOD (0.25 ng/mL)
PFPeA	3,247 (4.9)	63,652 (95.2)	0 (0.0)
PFHxA	35,574 (53.2)	31,326 (46.8)	0 (0.0)
PFHS	65,499 (97.9)	1,400 (2.1)	1,400 (2.1)
PFHpA	25,095 (37.5)	41,804 (62.5)	0 (0.0)
PFOA	66,857 (99.9)	42 (0.1)	42 (0.1)
PFOS	66,600 (99.6)	299 (0.5)	299 (0.5)
PFNA	65,348 (97.7)	1,551 (2.3)	1,551 (2.3)
PFDA	30,996 (46.3)	35,903 (53.7)	0 (0.0)
PFUnA	5,835 (8.7)	61,064 (91.3)	0 (0.0)
PFDoA	488 (0.7)	66,411 (99.3)	0 (0.0)

Denominator is total possible samples (66,899).

**Table 3:** Population serum concentrations for seven PFCs (ng/mL).

Age/sex	Measure	PFHxA^a^	PFHxA^b^	PFHS	PFHpA^a^	PFHpA^b^	PFOA	PFOS	PFNA	PFDA^a^	PFDA^b^
< 12 years											
Female	Mean	0.9	1.3	10.6	1.1	1.4	73.0	22.6	1.9	0.5	0.7
	Median	0.7	1.0	6.1	0.7	1.0	30.7	19.9	1.6	0.5	0.6
	Geometric mean	0.7	1.1	6.5	0.7	1.1	34.8	19.7	1.7	0.4	0.7
	SD	0.9	1.0	13.0	1.3	1.5	120.1	12.7	1.4	0.3	0.2

Male	Mean	1.0	1.3	12.6	1.1	1.5	82.1	24.6	1.9	0.5	0.7
	Median	0.7	1.0	6.9	0.7	1.0	35.1	21.7	1.6	0.5	0.6
	Geometric mean	0.7	1.1	7.4	0.7	1.1	39.1	21.5	1.7	0.4	0.7
	SD	1.0	1.0	18.3	1.3	1.5	129.1	13.4	1.1	0.3	0.3

Total	Mean	1.0	1.3	11.6	1.1	1.4	77.6	23.6	1.9	0.5	0.7
	Median	0.7	1.0	6.4	0.7	1.0	32.6	20.7	1.6	0.5	0.6
	Geometric mean	0.7	1.1	7.0	0.7	1.1	36.9	20.6	1.7	0.4	0.7
	SD	1.0	1.0	15.9	1.3	1.5	124.9	13.1	1.3	0.3	0.3

12–19 years											
Female	Mean	0.8	1.4	6.2	0.7	1.2	51.0	20.1	1.4	0.5	0.7
	Median	0.5	1.0	3.7	0.3	0.9	22.1	18.0	1.3	0.4	0.6
	Geometric mean	0.5	1.1	4.0	0.5	1.0	25.1	17.6	1.3	0.3	0.7
	SD	1.0	1.2	9.5	0.8	0.9	85.2	11.0	0.7	0.4	0.3

Male	Mean	0.9	1.4	8.3	1.0	1.5	68.4	23.4	1.6	0.3	0.7
	Median	0.6	1.0	4.9	0.6	1.0	30.2	20.5	1.5	0.5	0.7
	Geometric mean	0.6	1.1	5.3	0.6	1.2	33.9	20.6	1.5	0.3	0.7
	SD	1.3	1.5	11.8	1.2	1.4	104.9	12.7	0.8	0.4	0.3

Total	Mean	0.9	1.4	7.3	0.9	1.3	59.9	21.8	1.5	0.3	0.7
	Median	0.6	1.0	4.3	0.6	0.9	25.7	19.3	1.4	0.5	0.6
	Geometric mean	0.6	1.1	4.6	0.6	1.1	29.3	19.1	1.4	0.3	0.7
	SD	1.2	1.4	10.8	1.0	1.2	96.2	12.0	0.7	0.4	0.3

20–39 years											
Female	Mean	0.8	1.4	3.0	0.5	1.0	42.3	16.6	1.4	0.3	0.8
	Median	0.3	1.0	2.2	0.3	0.8	17.0	14.8	1.2	0.5	0.6
	Geometric mean	0.5	1.1	2.2	0.4	0.9	19.8	14.0	1.2	0.3	0.7
	SD	1.0	1.2	3.0	0.6	0.8	118.3	9.7	0.8	0.4	0.4

Male	Mean	1.0	1.5	5.3	0.6	1.2	76.5	24.3	1.7	0.4	0.8
	Median	0.6	1.0	3.8	0.3	0.8	28.3	22.2	1.6	0.5	0.7
	Geometric mean	0.6	1.2	3.8	0.4	1.0	33.4	20.7	1.6	0.5	0.7
	SD	1.4	1.6	7.6	0.7	1.0	208.1	12.8	0.8	0.4	0.8

Total	Mean	0.9	1.4	4.0	0.5	1.1	58.1	20.1	1.5	0.6	0.8
	Median	0.5	1.0	2.9	0.3	0.8	21.8	18.1	1.4	0.5	0.7
	Geometric mean	0.6	1.2	2.9	0.4	0.9	25.2	16.8	1.4	0.3	0.7
	SD	1.2	1.4	5.7	0.7	0.9	166.6	11.9	0.8	0.4	0.6

40–59 years											
Female	Mean	0.8	1.4	3.3	0.5	1.1	80.2	20.8	1.5	0.5	0.8
	Median	0.5	1.0	2.4	0.3	0.8	25.7	17.7	1.3	0.3	0.7
	Geometric mean	0.5	1.1	2.3	0.4	0.9	30.4	16.9	1.3	0.4	0.7
	SD	1.0	1.1	3.7	0.8	1.2	260.7	14.2	0.8	0.4	0.4

Male	Mean	0.9	1.4	4.7	0.6	1.2	120.4	26.6	1.6	0.5	0.8
	Median	0.5	1.0	3.4	0.3	0.8	37.7	23.5	1.5	0.3	0.7
	Geometric mean	0.6	1.2	3.5	0.4	1.0	43.8	22.3	1.5	0.4	0.7
	SD	1.1	1.2	16.8	0.8	1.2	339.0	16.7	0.9	0.8	1.1

Total	Mean	0.9	1.4	4.0	0.5	1.2	99.4	23.6	1.6	0.5	0.8
	Median	0.5	1.0	2.9	0.3	0.8	30.7	20.5	1.4	0.3	0.7
	Geometric mean	0.6	1.1	2.8	0.4	0.9	36.2	19.3	1.4	0.4	0.7
	SD	1.0	1.2	11.9	0.8	1.2	301.4	15.7	0.9	0.6	0.9

≥ 60 years											
Female	Mean	0.8	1.3	4.7	0.7	1.4	107.0	28.2	1.5	0.5	0.8
	Median	0.3	1.0	3.3	0.3	0.9	41.0	24.2	1.3	0.3	0.7
	Geometric mean	0.5	1.1	3.4	0.4	1.0	44.2	22.9	1.3	0.4	0.7
	SD	0.9	1.0	5.3	1.1	1.6	199.9	19.7	0.8	0.4	0.4

Male	Mean	0.9	1.5	4.6	0.6	1.3	120.8	30.3	1.5	0.5	0.8
	Median	0.3	1.0	3.4	0.3	0.9	42.8	26.1	1.4	0.3	0.7
	Geometric mean	0.5	1.2	3.4	0.4	1.0	47.2	24.7	1.3	0.4	0.7
	SD	1.1	1.4	5.3	0.9	1.3	394.1	23.2	0.9	0.4	0.5

Total	Mean	0.8	1.4	4.7	0.6	1.3	113.8	29.2	1.5	0.5	0.8
	Median	0.3	1.0	3.3	0.3	0.9	41.9	25.1	1.4	0.3	0.7
	Geometric mean	0.5	1.1	3.4	0.4	1.0	45.7	23.7	1.3	0.4	0.7
	SD	1.0	1.2	5.3	1.0	1.4	311.7	21.5	0.8	0.4	0.5

Total population											
Female	Mean	0.8	1.4	4.3	0.6	1.2	68.8	20.7	1.5	0.5	0.8
	Median	0.5	1.0	2.7	0.3	0.8	23.6	17.6	1.3	0.3	0.7
	Geometric mean	0.5	1.1	2.8	0.4	1.0	27.9	17.0	1.3	0.4	0.7
	SD	1.0	1.1	6.2	0.8	1.2	190.6	14.1	0.8	0.4	0.4

Male	Mean	0.9	1.4	5.9	0.7	1.3	98.2	26.0	1.7	0.5	0.8
	Median	0.6	1.0	3.8	0.3	0.9	33.7	22.9	1.5	0.3	0.7
	Geometric mean	0.6	1.2	4.0	0.4	1.0	39.4	21.9	1.5	0.4	0.7
	SD	1.2	1.4	12.8	0.9	1.3	284.3	16.5	0.9	0.6	0.8

Total	Mean	0.9	1.4	5.1	0.6	1.2	82.9	23.3	1.6	0.5	0.8
	Median	0.5	1.0	3.2	0.3	0.9	28.2	20.2	1.4	0.3	0.7
	Geometric mean	0.6	1.1	3.3	0.4	1.0	32.9	19.2	1.4	0.4	0.7
	SD	1.1	1.3	10.0	0.9	1.2	240.8	15.6	0.9	0.5	0.7

a Substitution with 50% of LOD for values reported as < LOD.

b No substitution for values reported as < LOD.

**Table 4:** Age- and sex-adjusted serum PFC concentrations by water district (ng/mL).

Water district	PFHxA^a^	PFHxA^b^	PFHS	PFHpA^a^	PFHpA^b^	PFOA	PFOS	PFNA	PFDA^a^	PFDA^b^
City of Belpre (Ohio)										
Mean	0.86	1.41	5.82	0.64	1.10	42.96	23.18	1.50	0.47	0.73
SE	0.01	0.02	0.11	0.01	0.02	2.48	0.16	0.01	0.01	0.01

Little Hocking Water Association (Ohio)										
Mean	0.85	1.39	5.70	1.15	1.86	227.59	23.47	1.60	0.50	0.77
SE	0.01	0.02	0.09	0.01	0.01	2.03	0.14	0.01	0.01	0.01

Lubeck Public Service District (West Virginia)										
Mean	1.02	1.51	5.58	0.61	1.05	92.36	24.96	1.64	0.55	0.82
SE	0.01	0.01	0.08	0.01	0.01	1.78	0.12	0.01	0.00	0.01

Mason County (West Virginia)										
Mean	0.72	1.28	4.15	0.38	0.83	16.00	23.01	1.59	0.45	0.72
SE	0.01	0.02	0.09	0.01	0.02	2.06	0.14	0.01	0.01	0.01

Tuppers Plains (Ohio)										
Mean	0.84	1.38	4.48	0.43	0.91	42.07	22.29	1.50	0.53	0.74
SE	0.01	0.02	0.08	0.01	0.02	1.96	0.13	0.01	0.04	0.01

Village of Pomeroy (Ohio)										
Mean	0.83	1.38	4.25	0.38	0.83	15.96	20.97	1.46	0.47	0.69
SE	0.02	0.03	0.17	0.01	0.04	3.83	0.25	0.02	0.00	0.02

Private Well (West Virginia or Ohio)										
Mean	0.65	1.88	9.27	0.82	1.48	132.56	26.15	1.67	0.40	0.81
SE	0.09	0.16	0.79	0.07	0.14	18.41	1.22	0.07	0.01	0.08

a Substitution with 50% of LOD for values reported as < LOD.

b No substitution for values reported as < LOD.

**Table 5:** Summary of intra- and interlab quality assurance.

	Intralab comparisons^a^					Interlab comparisons^b^				
Compound/measure	Primary lab test results (ng/mL)	Primary lab QA sample (ng/mL)	Absolute difference	Percent difference	Coefficient of variation	Primary lab test results (ng/mL)	Secondary lab QA sample (ng/mL)	Absolute difference	Percent difference	Coefficient of variation
PFHxA										
No. of samples	664	612	574	574	574	1,180	All values	NA	NA	NA
Mean	1.3	1.3	0.3	18.3%	0.1	1.2	reported as			
Median	1.0	0.9	0.1	11.8%	0.1	0.9	< LOD			

PFHS										
No. of samples	1,236	1,241	1,234	1,234	1,234	2,561	2,319	2,316	2,316	2,316
Mean	4.6	4.6	0.4	9.4%	0.1	4.9	4.6	1.3	28.8%	0.2
Median	3.1	3.1	0.2	4.9%	0.0	3.5	3.2	0.8	23.9%	0.2

PFHpA										
No. of samples	446	434	410	410	410	1,074	583	572	572	572
Mean	1.2	1.2	0.1	8.5%	0.1	1.5	1.4	0.8	44.3%	0.3
Median	0.9	0.9	0.1	4.2%	0.0	1.0	0.9	0.5	42.0%	0.3

PFOA										
No. of samples	1,269	1,269	1,269	1,269	1,269	2,603	2,599	2,599	2,599	2,599
Mean	77.3	80.4	9.7	10.1%	0.1	134.5	129.7	27.1	21.0%	0.2
Median	25.3	25.3	1.4	5.2%	0.0	43.9	39.3	6.9	16.7%	0.1

PFOS										
No. of samples	1,261	1,261	1,260	1,260	1,260	2,594	2,588	2,588	2,588	2,588
Mean	22.9	23.2	2.0	8.4%	0.1	22.3	22.7	3.9	17.4%	0.1
Median	19.5	19.8	0.7	4.1%	0.0	19.4	19.6	2.6	14.0%	0.1

PFNA										
No. of samples	1,246	1,243	1,240	1,240	1,240	2,539	2,329	2,314	2,314	2,314
Mean	1.6	1.6	0.2	9.0%	0.1	1.6	1.3	0.4	28.7%	0.2
Median	1.4	1.4	0.1	6.5%	0.1	1.4	1.2	0.3	24.9%	0.2

PFDA										
No. of samples	566	570	516	516	516	1,200	409	371	371	371
Mean	0.8	0.8	0.1	6.9%	0.1	0.8	0.8	0.3	33.8%	0.2
Median	0.7	0.7	0.0	0.0%	0.0	0.7	0.6	0.2	28.6%	0.2

NA, not applicable; QA, quality assurance.

a Comparison between matched samples for main test results and blinded, duplicate quality assurance samples sent to the primary lab.

b Comparison between matched samples for main test results and blinded, duplicate quality assurance samples sent to the secondary lab.
